# Carbon-Nanotube-Based Superhydrophobic Magnetic Nanomaterial as Absorbent for Rapid and Efficient Oil/Water Separation

**DOI:** 10.3390/nano14231942

**Published:** 2024-12-03

**Authors:** Rabiga M. Kudaibergenova, Fernanda F. Roman, Adriano S. Silva, Gulnar K. Sugurbekova

**Affiliations:** 1 Department of Chemistry and Chemical Technology, Faculty of Technology, M. Kh. Dulaty Taraz University, 60 Tole Bi Street, Taraz 080000, Kazakhstan; 2 CIMO, LA SusTEC, Instituto Politécnico de Bragança, Campus de Santa Apolónia, 5300-253 Bragança, Portugal; adriano.santossilva@ipb.pt; 3 Department of Chemistry, Faculty of Natural Sciences, Eurasian National University Named after L.N. Gumilyov, Astana 010000, Kazakhstan; sugurbekova.g55@gmail.com

**Keywords:** superhydrophobic, magnetic, polyurethane sponge, CNT, oil/water separation

## Abstract

In this work, the simple fabrication of a new superhydrophobic magnetic sponge based on CNTs, NiFe_2_O_4_ nanoparticles, and PDMS was investigated. CNTs were synthesized by chemical vapor deposition (CVD) on a nickel ferrite catalyst supported on aluminum oxide (NiFe_2_O_4_/Al_2_O_3_). The synthesis of nickel ferrite (NiFe) was accomplished using the sol–gel method, yielding magnetic nanoparticles (43 Am^2^kg^−1^, coercivity of 93 Oe, 21–29 nm). A new superhydrophobic magnetic PU/CNT/NiFe_2_O_4_/PDMS sponge was fabricated using a polyurethane (PU) sponge, CNTs, NiFe_2_O_4_ nanoparticles, and polydimethylsiloxane (PDMS) through the immersion coating method. The new PU/CNT/NiFe_2_O_4_/PDMS sponge exhibits excellent superhydrophobic/oleophilic/mechanical properties and water repellency (water absorption rate of 0.4%) while having good absorption of oil, olive oil, and organic liquids of different densities (absorption capacity of 21.38 to 44.83 g/g), excellent separation efficiency (up to 99.81%), the ability to be reused for removing oil and organic solvents for more than 10 cycles, and easy control and separation from water using a magnet. The new PU/CNT/NiFe_2_O_4_/PDMS sponge is a promising candidate as a reusable sorbent for collecting oil and organic pollutants and can also be used as a hydrophobic filter due to its excellent mechanical properties.

## 1. Introduction

The treatment of oily wastewater has become an important global issue in the fight against environmental pollution resulting from oil leaks and spills [1,2]. There has been an increase in oil production and transportation by sea, as well as oil spills, leading to environmental disasters affecting marine ecology [3]. Oil spills that reach the sea surface are easily spread over large areas by waves and wind, and therefore require rapid intervention. Therefore, currently, wastewater treatment from oil and petroleum products and organic solvents is a very hot topic. To solve these problems, various methods of separating oil and water, as well as new materials, are currently being studied [4,5]. Porous materials such as sponges, fabrics, and membranes have become ideal substrates for the preparation of superhydrophobic, oleophilic materials due to their low cost, abundant sources, elasticity, and high and efficient absorption capacity [6,7,8]. Yang et al. prepared a robust superhydrophobic carbon fiber cotton fabric with a rough surface structure and low surface energy, which enabled the cotton fabric to exhibit superhydrophobic/superoleophilic and self-cleaning properties, effectively separating various oil/water mixtures and emulsions [9]. Zhang et al. prepared a graphene-based superhydrophobic sponge that exhibits excellent absorption capacity, exceeding up to 90 times its own weight [10]. Deng et al. proposed a simple and cost-effective method for the preparation of superhydrophobic melamine sponges, which exhibited stable superhydrophobicity with a maximum water contact angle of 158° [11]. The sponges showed outstanding absorption capacity and excellent mechanical properties for the separation of stratified oil/water mixtures by repeated absorption–extrusion processes with a separation efficiency of nearly 100%. Liu et al. developed an environmentally friendly superhydrophobic polyurethane sponge, which has excellent separation properties of oil/water mixtures or emulsions and high oil absorption capacity (20–55 g/g) for various kinds of oils, using PDMS and dehydroabietic acid grafted with Al_2_O_3_ nanoparticles via the dipping method [12]. Rather et al. [13] synthesized superhydrophobic cotton that is capable of completely repelling water both in air and under oil, highly selective absorption with efficiency of more than 2000 wt.% for both heavy and light oil, and active filtration of oil under gravity. Shome et al. [14,15] fabricated a physically/chemically robust superhydrophobic material that did not lose its superhydrophobic properties even after exposure to various aggressive physical and chemical abrasives, as well as UV irradiation.

The application areas of magnetic nanoparticles functionalized with various surfactants are varied. A number of authors have demonstrated their effective use in catalysis [16], in medicine [17], and especially as highly effective adsorbents for removing pollutants from wastewater. The authors of [18] synthesized amino-functionalized core–shell Fe_3_O_4_@SiO_2_ magnetic nanoparticles, which showed high adsorption properties towards methylene blue, Congo red, and eosin. Y. Guo et al. [19] prepared an amino-functionalized CoFe_2_O_4_/SBA–15 (NH_2_–CoFe_2_O_4_/SBA–15) nanocomposite for the effective removal of Pb^2+^ from aqueous solution. The adsorbent based on SBA–15 and magnetic CoFe_2_O_4_ was ffectively separated from water using an external magnetic field. Also, the authors of [20] developed a superhydrophobic magnetic nanomaterial based on reduced graphene oxide, MgFe_2_O_4_ nanoparticles, and PDMS, which had highly effective separation properties for oil and organic solvent/water mixtures and could be moved to the contaminated water zone using a magnet.

Among various superhydrophobic materials, CNT-coated absorbents have unique superhydrophobic properties due to their chemical stability and hydrophobic interactions [21]. In addition, CNTs can be easily synthesized using waste materials, such as plastic waste [22], contributing to achieving the circular economy goals. In this work, a new CNT-based superhydrophobic magnetic nanomaterial suitable for recycling was prepared for the selective sorption of oil/organic solvents from wastewater. First, the synthesis and study of the physicochemical characteristics of CNTs and NiFe_2_O_4_ magnetic nanoparticles were carried out. Second, the authors performed a facile method for the preparation of a new superhydrophobic magnetic material through immersion coating using a polyurethane (PU) sponge containing CNTs, NiFe_2_O_4_ magnetic nanoparticles, and polydimethylsiloxane (PDMS). The obtained superhydrophobic/super oleophilic sponge has excellent potential for practical application due to its ability to rapidly and efficiently absorb oil within a few seconds, possessing excellent selectivity for oil and organic solvents.

## 2. Materials and Methods

### 2.1. Chemicals and Apparatus

Alumina was obtained from BASF (Darmstadt, Germany) in the form of pellets. Before being used, it was ground and sieved in the 53–106 μm range.

High-density polyethylene (HDPE, melt index 2.2 g/10 min), low-density polyethylene (LDPE, weight-average molecular weight ~35,000 g mol^−1^, number-average molecular weight ~7700), polypropylene (PP, weight-average molecular weight ~250,000 g mol^−1^, number-average molecular weight ~67,000), and ethylene glycol (HOCH_2_CH_2_OH, 99.5 wt%) were obtained from Sigma-Aldrich (St. Louis, MI, USA).

Iron (III) nitrate nonahydrate (Fe(NO_3_)_3_·9H_2_O, 99%), iron (III) chloride hexahydrate (FeCl_3_·6H_2_O, 98%), and ammonia (NH_4_OH, 28–30% in H_2_O) were purchased from Merk (Darmstadt, Germany).

Nickel (II) nitrate hexahydrate (Ni(NO_3_)_2_·6H_2_O, 99%) was obtained from VWR Chemicals (Radnor, PA, USA).

Ethanol absolute (CH_3_CH_2_OH, 96%) and nickel (II) chloride hexahydrate (NiCl_2_·6H_2_O, 98%) were supplied by Fisher Chemicals (Waltham, MA, USA).

Hexane (for HPLC, ≥97.0%) was purchased from Sigma-Aldrich (St. Louis, MI, USA). The polyurethane sponge was purchased at a hardware store (density 22 kg/m^3^). Polydimethylsiloxane was hydride-terminated (PDMS, average Mn—24,000).

The NiFe and CNT-CS samples were analyzed through X-ray diffraction (XRD) using a PANalyticalX’Pert PRO X-ray diffractometer equipped with an X’Celerator detector and a secondary monochromator (Cu Kα λ = 0.154 nm). XRD data from NiFe were used to estimate crystallite size according to the Halder–Wagner and size–strain plot mathematical models, as reported elsewhere [23]. CNT-CS morphology was examined through transmission electron microscopy (TEM). TEM images were taken with a JEOL JEM 2100 (Jeol, Akishima, Tokyo, Japan), operating at 200 kV. Prior to the TEM analysis, CNT-CS was washed with 50% H_2_SO_4_ (140 °C, 3 h) to remove any interference from the metallic particles during TEM analysis. Fourier Transform Infrared Spectroscopy (FT-IR) was performed in a PerkinElmer FT-IR spectrophotometer UATR Two (PerkinElmer, Inc., Waltham, MA, USA) with a resolution of 4 cm^−1^ and a scan range of 4000 to 450 cm^−1^. For the FT-IR analysis, each sample was prepared by mixing ca. 1 mg of nanomaterial and 100 mg of KBr, pressed in a pellet. The superparamagnetic behavior of NiFe was verified with a superconducting quantum interference device (SQUID-VSM) magnetometer (Quantum Design Inc., San Diego, CA, USA), recording the hysteresis curve at 300 K, with a magnetic field varying from −20 to 20 kOe. N_2_ adsorption/desorption isotherms were recorded in a Quantachrome NOVA Touch (Quantachrome GmbH & Co. KG, Odelzhausen, Germany) adsorption analyzer at −196 °C to determine the Brunauer–Emmett–Teller specific surface area (S_BET_) [24].

The morphology of the CNT, NiFe samples, and newly prepared PU/CNT/NiFe_2_O_4_/PDMS sponge were characterized using a Carl Zeiss Crossbeam 540 with a GEMINI II scanning electron microscope (GmbH, Jena, Germany), and the water contact angles (CA) of the PU/CNT/NiFe_2_O_4_/PDMS sponge were measured using a Dataphysics Instrument OCA 15EC system (GmbH, Filderstadt, Germany).

### 2.2. Synthesis of Magnetic Nickel Ferrite Nanoparticles

The synthesis of magnetic nickel ferrite (NiFe) nanosized cores was performed through a sol–gel method following the protocol of Giannakopoulou et al. [25]. Briefly, 10 mL of a 1 M Ni(NO_3_)_2_·6H_2_O solution prepared in ethanol was mixed with 40 mL of a 0.5 M solution of Fe(NO_3_)_3_·9H_2_O and ethylene glycol. Afterwards, the solution was stirred at 60 °C for 2 h to sustain the transition from the sol to the gel state. Finally, the temperature was increased to 220 °C until the complete drying of the gel. To reach the proper crystal structure, the dark gray powder obtained underwent a high-temperature treatment (12 h at 300 °C, 24 h at 600 °C, and 1 h at 1000 °C at a heating ramp of 120 °C min^−1^), resulting in the NiFe sample.

### 2.3. Synthesis of Nickel Ferrite Supported over Alumina for CNT Growth Using CVD

The synthesis of the CVD catalyst was achieved through co-precipitation [26] in the presence of the support. NiCl_2_·6H_2_O and FeCl_3_.6H_2_O were dissolved in water (200 mL) along with Al_2_O_3_. The masses were calculated to achieve a deposition of 20% of the active phase over the support. The salts and alumina were vigorously stirred (600 rpm) for 60 min to ensure proper dispersion. Then, precipitation was forced through the dropwise addition of NH_4_(OH) (1 mol L^−1^) using a peristaltic pump until pH 9 was reached. The precipitated particles were further stirred for 60 min and then left undisturbed to precipitate in the bottom of the reaction vessel. Then, the particles were separated and washed with water until a neutral pH was reached, following by drying overnight 60 °C. The obtained material was heat-treated at 850 °C for 3 h, leading to NiFe_2_O_4_/Al_2_O_3_.

### 2.4. Synthesis of CNTs

The CNTs were synthesized via chemical vapor deposition (CVD) over a nickel ferrite-based catalyst supported in alumina (NiFe_2_O_4_/Al_2_O_3_). The CVD process occurred in a vertical oven (TH/TV, Termolab, Águeda, Portugal), as displayed elsewhere [27]. A mixture of three polyolefins (LDPE, HDPE, and PP) were considered as the carbon source. The mixture of three polymers consisted of 35:25:40 of LDPE/HDPE/PP (on an mass basis). One gram of the CVD catalyst was loaded in the lower region of the oven, and 5 g of the plastic mixture was loaded in the upper region of the oven, as shown in Figure S1 in the Supplementary File. The polymer and catalyst were loaded in the upper and lower regions, respectively, to allow the nitrogen gas to carry the carbon-rich gas fraction resulting from polymer cracking to the catalyst particles. The synthesis was carried out at 850 °C for 30 min, under a nitrogen flow (50 mL min^−1^). The obtained CNTs were named CNT-CS.

### 2.5. Preparation of Superhydrophobic Magnetic PU Sponges

Superhydrophobic magnetic sponges were developed through the immersion coating method [20,28] using a PU sponge, CNT, NiFe_2_O_4_ nanoparticles, and PDMS. For this purpose, the PU sponge was cut into pieces (size 1.5 × 1.5 × 1 cm) and thoroughly washed in ultrasonic acetone, then in distilled water, for 30 min, and dried at 60 °C. The cleaned pieces of polyurethane sponge were modified by immersing them in a 50 mL suspension of NiFe_2_O_4_ nanoparticles (130 mg), CNTs (40 mg), and PDMS (80 mg) in hexane, and treated with ultrasound for 7 h. Afterward, the treated sponge pieces were washed several times with distilled water and dried at 60 °C. The obtained superhydrophobic magnetic sponge was named PU/CNT/NiFe_2_O_4_/PDMS.

### 2.6. Study of Superhydrophobic and Oleophilic Properties

To study the hydrophobic property of the prepared PU/CNT/NiFe_2_O_4_/PDMS sponge, a 1.5 × 1.5 × 1 cm piece was dipped into distilled water at room temperature for 10 min with tweezers. The weight of the sponge was measured before and after immersion to calculate the water uptake by weight. All experiments were performed three times.

### 2.7. Study of Hydrophobic Properties, Oil/Water Separation Efficiency, and Oil Absorption Capacity

The hydrophobic properties of the initial PU sponge and the prepared PU/CNT/NiFe_2_O_4_/PDMS sponge were studied. For each sponge, a 1.5 × 1.5 × 1 cm piece with a known weight was immersed in distilled water (100 mL) at room temperature for 10 min by applying an external force with tweezers. The mass of each sponge was weighed three times before and after immersion to calculate the mass absorption of water. All experiments were performed in triplicate. The superhydrophobic properties of the initial PU sponge and the new magnetic sponge were investigated from the point of view of measurements of water absorption over the entire surface of the resulting sponge

To study the separation efficiency of various mixtures of oil, organic solvents, and water, the prepared PU/CNT/NiFe_2_O_4_/PDMS sponge was immersed in a mixture of oil, organic solvent, and water with a known composition until the organic liquids were completely absorbed. Then, the sponge was taken out of the water to check the amount of oil or organic liquids remaining in the water. The separation efficiency of the prepared superhydrophobic sponge for various mixtures of oil, organic solvents, and water was calculated by measuring the amount of oil and organic liquids remaining in the water before and after absorption using Equation (1):

(1) $$R_{\left(\%\right)}=\left[\frac{C_{2}-C_{1}}{C_{0}}\right]\times 100\%,$$

In Formula (1), R is the separation efficiency, %; C_2_ and C_1_ are the total amount of oil/water mixed before and after separation; and C_0_ is the amount of oil before absorption. The separation experiments were performed three times, and the average experimental value was obtained.

To study the absorption capacity, the modified sponge was immersed in oil or an organic solvent for 10 s. After that, the PU/CNT/NiFe_2_O_4_/PDMS sponge was weighed three times before and after immersion to determine the average weight. The absorption capacity was calculated based on the amount of absorbed oil according to Equation (2):

(2) $$Q_{\left(\frac{g}{g}\right)}=\frac{m_{2}-m_{1}}{m_{1}},$$

In Formula (2), Q is the absorption capacity, g/g; m_1_ is the mass of the PU/CNT/NiFe_2_O_4_/PDMS, g; and m_2_ is the mass of the PU/CNT/NiFe_2_O_4_/PDMS after oil absorption.

## 3. Results and Discussion

### 3.1. Structural, Morphological, and Physicochemical Characterization of CNT and NiFe_2_O_4_ Nanoparticles

#### 3.1.1. FTIR

**Figure 1:**
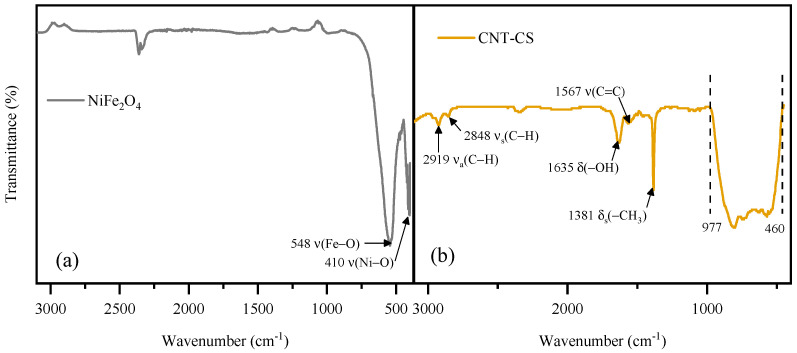
FTIR spectra of (**a**) NiFe_2_O_4_ and (**b**) CNT-CS.

The FTIR spectra of the samples are displayed in Figure 1. The band located at ~548 cm^−1^ can be ascribed to the tetrahedral sites of Fe-O bonds [29], and the bands located at lower wavenumbers (ca. 410 cm^−1^) can be associated with Ni-O stretching vibration [30]. The characteristic band of CNTs (i.e., stretching vibration of C=C bonds [31]) was detected at 1567 cm^−1^ for CNT-CS, indicating the presence of sp^2^ bonds, which is characteristic of graphene layers. Additionally, the presence of -CH_2_ groups, likely linked to defects [32], was identified based on the bands located at 2919 and 2848 cm^−1^ (stretching vibrations of C-H bond in CH_2_ group) and at 1381 cm^−1^ (symmetrical bending of -CH_2_ or -CH_3_ groups) [31]. The presence of these bands is associated with defects, giving rise to sp^3^ bonds between the carbon atoms. The samples also exhibited bands between 977 and 460 cm^−1^, related to the catalyst used for CNT growth. The remaining bands (1635 cm^−1^) were ascribed to adsorbed water [33].

#### 3.1.2. XRD

**Figure 2:**
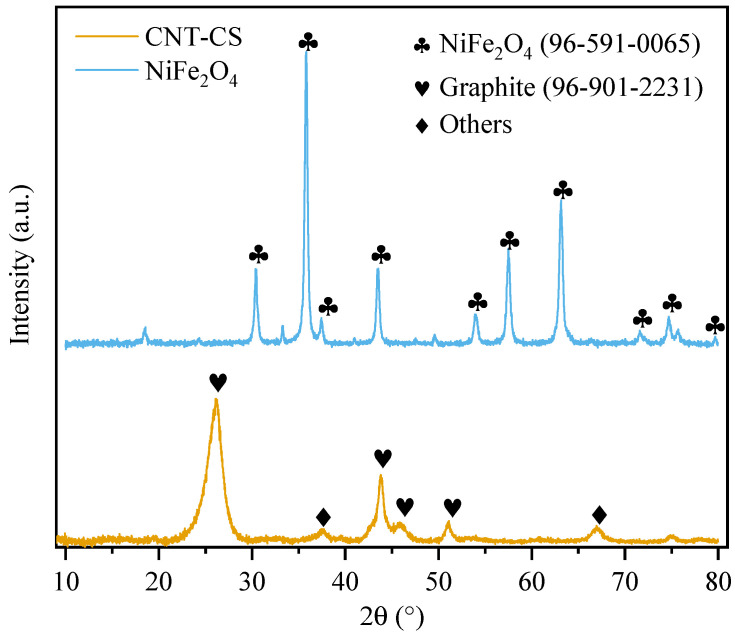
X-ray diffractogram of CNT-CS and NiFe_2_O_4_.

The X-ray diffractogram is displayed in Figure 2. The characteristic crystal structure of the nickel ferrite (inverse spinel with a face-centered cubic arrangement) was confirmed using the NiFe_2_O_4_ reference card (96-591-0065) from the Crystallography Open Database (COD). Previous studies have reported similar diffractograms for NiFe_2_O_4_ particles [23]. The crystallite sizes were estimated to be 21 and 29 nm according to the Halder–Wagner and size–strain methods, respectively, confirming the formation of nanoparticles. These values are similar to previously reported values for nickel ferrite nanoparticles synthesized using sol–gel [34,35], as sol–gel is a technique that allows fine control over the size and distribution of particle diameters [36]. The equations, methodology, and linear regression used to determine the crystallite sizes are fully described in the Supplementary Materials [37] (Text S1 and Figure S1).

The CNT-CS sample displayed typical peaks associated with graphite (26°, 43°, 45° and 51°), according to COD card 96-901-2231. The remaining peaks in CNT-CS (37° and 66°) could be associated with magnetic metallic phases attached to the CNT structure, such as cementite, as previously reported [27]. Other works reported similar diffractogram patterns for CNTs [38,39].

#### 3.1.3. SEM and TEM Images

**Figure 3:**
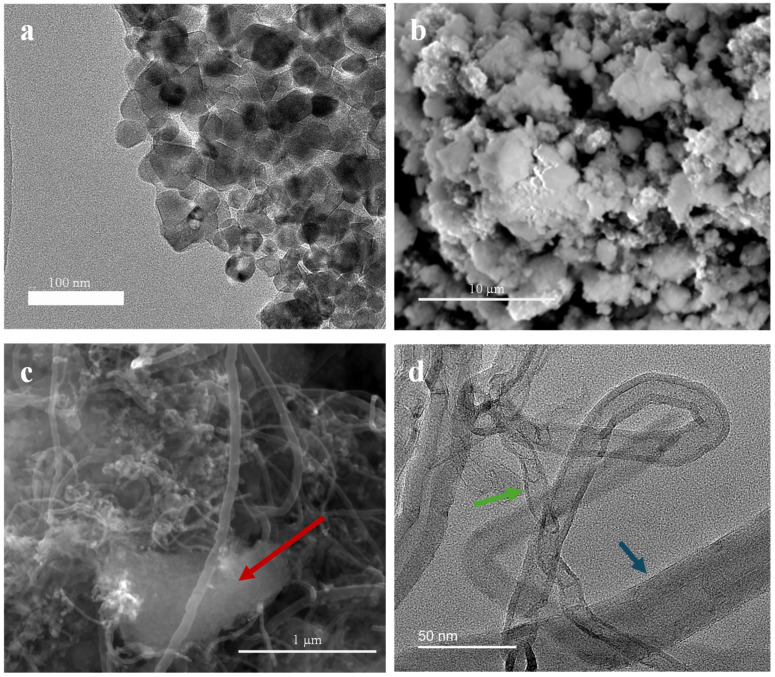
TEM of (**a**) NiFe_2_O_4_, (**b**,**c**) SEM, and (**d**) TEM of CNT-CS.

The morphology of the materials synthesized in this work was assessed via scanning and transmission electron microscopy (Figure 3). NiFe_2_O_4_ showed sizes within a narrow range, which is related to the synthesis procedure that ensures nanoparticles have a monodispersive size distribution. The average nanoparticle size determined by TEM was 22 ± 4 nm, which confirms the formation of nanoparticles and is about the same as that determined by the mathematical models Halder–Wagner and the size–strain plot applied to the XRD result. Still, Halder–Wagner showed more precision in finding the nanoparticle size, with an error smaller than 1%. Other works also reported Halder–Wagner as the optimal method to determine particle sizes using XRD results in single-phase inorganic materials [40].

A CNT was successfully obtained, as seen in the SEM and TEM pictures of CNT-CS (Figure 3c,d). It is possible to see the formation of tubular structures (Figure 3c,d) with an open cavity (Figure 3d), both with straight walls (indicated by the blue arrow, Figure 3d) and curly/defective walls (indicated by the green arrow, Figure 3d). Particles from the catalyst used to grow the nanostructures (red arrow, Figure 3c) can also be seen. CNT-CS resulted in an outer diameter in the range of 7–38 nm, with some CNTs displaying 5–10 walls and others with over 30 walls. Similar results have been reported for CNTs grown from polymers [27,41,42].

#### 3.1.4. Magnetic Characterization

**Figure 4:**
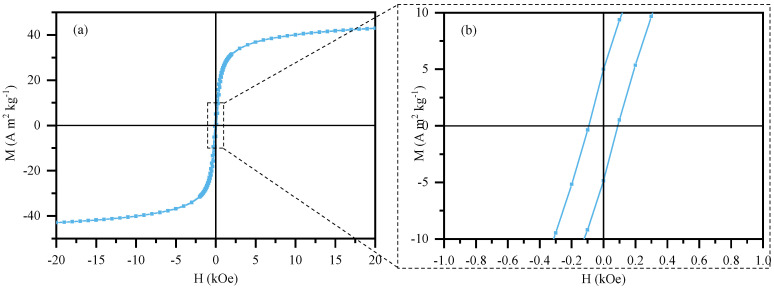
(**a**) Magnetic response of NiFe_2_O_4_ at 300 K and (**b**) close-up of the center region to show low coercivity and remanence.

The magnetic response of the nanoparticle NiFe_2_O_4_ was assessed at 300 K, and the mass-relative magnetization (M) as a function of the magnetic field (H) applied is shown in Figure 4. The NiFe nanoparticle resulted in saturation magnetization of approximately 43 Am^2^kg^−1^ and coercivity of 93 Oe. The remanence obtained for NiFe was 5 Am^2^kg^−1^. Similar results have previously been reported for NiFe nanoparticles synthesized via the sol–gel or solvo-thermal method [23,43]. The values found for the sample, verified using SQUID-VSM through the absence of hysteresis; rapid saturation in low fields; low coercivity (ca. 93 Oe); and low remanent magnetization (4.91 Am^−2^kg^−1^) enabled to classify the nanoparticle as superparamagnetic, which means the nanoparticles respond to magnetic fields when exposed to them, but return to their non-magnetic state once the magnetic field is removed [40]. The superparamagnetic behavior of NiFe is directly associated with the synthesis route: sol–gel is advantageous for synthesizing nanostructures, which allowed us to obtain NiFe nanoparticles below the critical size of 30 nm [44]. This characteristic is particularly interesting for liquid-phase applications considering how easy it is to recover the magnetic material without magnetizing the sample after the removal of the magnet.

**Figure 5:**
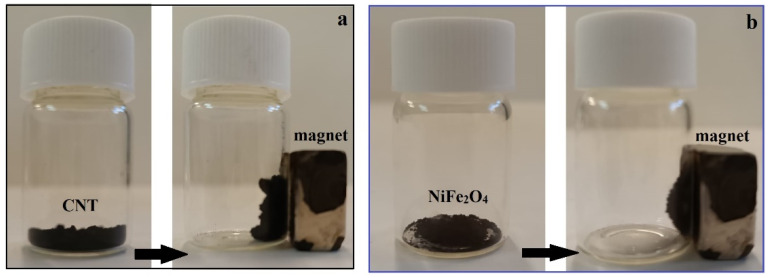
Magnetic properties of (**a**) CNT-CS and (**b**) NiFe_2_O_4_ nanoparticles.

The magnetic response of the materials (NiFe nanoparticles, CNT-CS, and sponge) was also demonstrated with a magnet (Figure 5a,b). For the newly prepared PU/CNT/NiFe_2_O_4_/PDMS sponge, see Supplementary Material Video S1. The magnetic properties were tested using a magnet, as shown in Figure 5a,b (and in Video S1). CNT, NiFe_2_O_4_ nanoparticles, and the newly prepared PU/CNT/NiFe_2_O_4_/PDMS sponge were perfectly attracted to the magnet. The magnetic properties of CNTs are due to the residual catalyst particles [45]. And the excellent magnetism of the new PU/CNT/NiFe_2_O_4_/PDMS sponge indicates that the magnetic NiFe_2_O_4_ nanoparticles and CNTs were successfully filled in the initial PU sponge.

### 3.2. Characterization of Newly Prepared PU/CNT/NiFe_2_O_4_/PDMS Sponge

#### 3.2.1. SEM Images of Newly Prepared PU/CNT/NiFe_2_O_4_/PDMS Sponge

**Figure 6:**
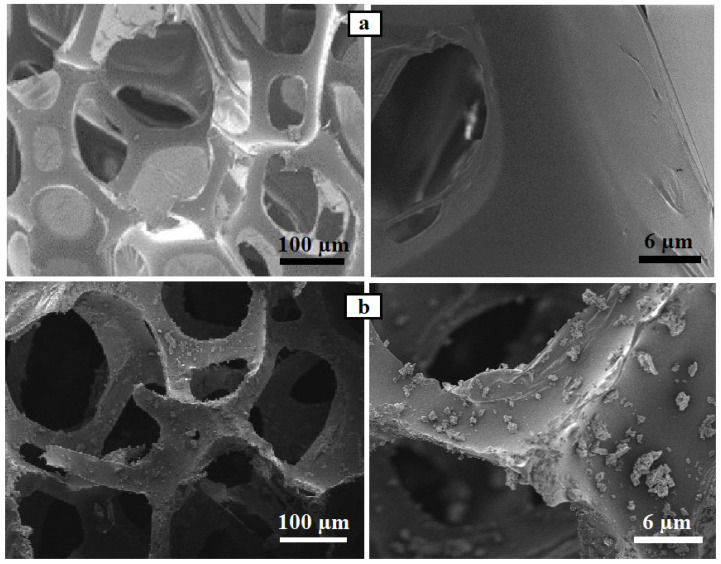
SEM images of (**a**) PU sponge and (**b**) PU/CNT/NiFe_2_O_4_/PDMS sponge.

The morphological characteristics of the newly prepared PU/CNT/NiFe_2_O_4_/PDMS sponge were studied using SEM. Figure 6 presents the SEM images of the initial PU sponge and prepared PU/CNT/NiFe_2_O_4_/PDMS sponge. From Figure 6a, it is clear that the sponge has a porous structure, which is very convenient for absorbing liquid. Also, the newly prepared PU/CNT/NiFe_2_O_4_/PDMS sponge has the same porous structure after immersion coating, which means that immersion does not destroy the porous structure of the sponge (Figure 6b). Also, from Figure 6, it is clearly seen that the initial PU sponge has a smooth and flat surface, while the newly prepared PU/CNT/NiFe_2_O_4_/PDMS sponge has an uneven, rough, and coarse structure [28]. The irregularities and roughness in the structure are of great significance to obtain an ideal hydrophobic and oleophilic surface. This clearly shows that the superhydrophobic/oleophilic magnetic nanoparticles of CNT, NiFe_2_O_4_, and PDMS are ideally and uniformly distributed over the entire surface of the polyurethane sponge. PDMS enhances the hydrophobic/oleophilic property and also acts as an adhesive, and CNT enables the sponge to have superhydrophobic/oleophilic property. Also, irregularities and roughness in the structure prevent water from entering the pores of the sponge, thereby improving the hydrophobic/oleophilic quality of the sponge.

#### 3.2.2. Hydrophobic and Oleophilic Properties of Newly Prepared PU/CNT/NiFe_2_O_4_/PDMS Sponge

**Figure 7:**
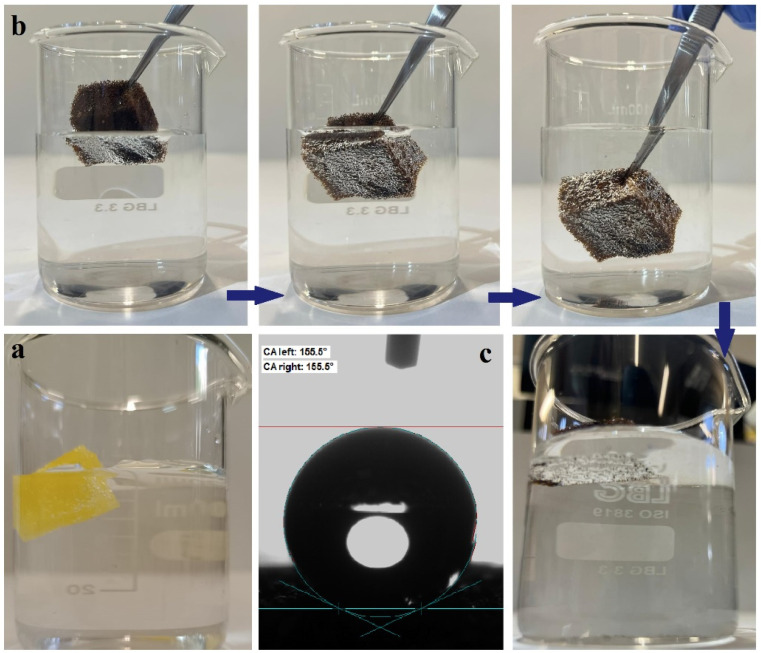
Photos of immersion in water: (**a**) the initial PU sponge; (**b**) demonstrating the silver mirror phenomenon of the newly prepared PU/CNT/NiFe_2_O_4_/PDMS sponge; (**c**) CA of the superhydrophobic sponge after immersion.

The hydrophobic properties of the PU/CNT/NiFe_2_O_4_/PDMS sponge were determined by immersing it in water at room temperature for 10 min. Figure 7 shows that the modified sponge, immersed in water, was held by force with tweezers, and it can also be seen that a silver mirror surface, typical of hydrophobic coatings, was formed between the surface of the sponge and the water. The hydrophobic properties were determined by calculating the percentage of absorbed water with the measurement of the sponge weight before and after immersion. Also, Figure 7 shows that after the immersion time, the initial PU sponge completely sank in the water (Figure 7a), and the newly prepared PU/CNT/NiFe_2_O_4_/PDMS sponge floated on the surface of the water (Figure 7b), which proves the hydrophobicity of the sponge. Experiments to determine the degree of hydrophobicity were carried out three times, and the results showed that the percentage of water absorption of the PU/CNT/NiFe_2_O_4_/PDMS sponge was 0.4%. The contact angle (CA) after immersion was 155.5° (Figure 7c) [46].

To demonstrate the hydrophobic and oleophilic properties of the newly prepared PU/CNT/NiFe_2_O_4_/PDMS sponge, water or gasoline droplets were applied to the surface. As shown in Video S2 in the Supplementary Materials, the water droplet on the sponge surface formed a ball shape and rolled off the surface easily (the roll-off angle was 0.3°), demonstrating superhydrophobic properties [46]. Conversely, drops of gasoline applied to the surface of the sponge were quickly absorbed (Video S3 in the Supplementary Material) and penetrated into the sponge (the contact angle with gasoline was 0°), which proves its excellent oleophilic qualities.

#### 3.2.3. Oil/Organic Solvent–Water Separation Efficiency and Oil/Organic Solvent Absorption Capacity

**Figure 8:**
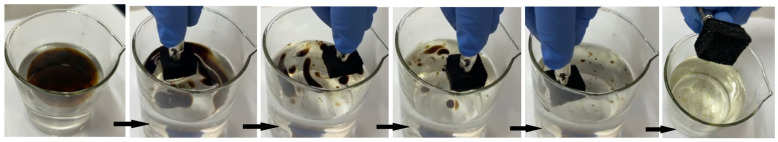
Photos of the process of removing crude oil from a crude oil/water mixture using a PU/CNT/NiFe_2_O_4_/PDMS sponge.

For the practical application of the sorbent for water treatment, determining its absorption and recycling abilities was very important. To determine these properties, several tests were conducted on the newly prepared PU/CNT/NiFe_2_O_4_/PDMS sponge. First, we tested the use of PU/CNT/NiFe_2_O_4_/PDMS for the selective collection of crude oil from the crude oil/water mixture. It is worth noting that the new PU/CNT/NiFe_2_O_4_/PDMS sponge completely and selectively absorbed crude oil from the water surface and repelled water (Figure 8), which indicates its high absorption capacity and superhydrophobicity. In addition, the new PU/CNT/NiFe_2_O_4_/PDMS sponge had high elasticity and toughness, and it could be flexibly moved and removed from the water surface by a magnet (Figure 8). Also, the recyclability of a new PU/CNT/NiFe_2_O_4_/PDMS sponge was investigated for the selective sorption of crude oil from crude oil-contaminated water through repeated absorption/desorption processes [29]. After repeated use (more than 10 cycles), the new PU/CNT/NiFe_2_O_4_/PDMS sponge maintained a flexible, elastic and durable structure, could be easily bent and compressed, and also retained its superhydrophobic, oleophilic, and magnetic properties.

**Figure 9:**
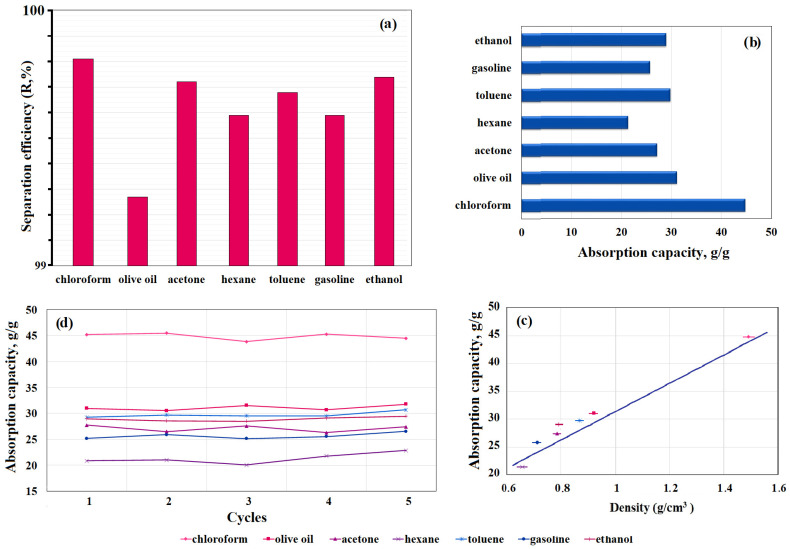
(**a**) Separation efficiency; (**b**) adsorption capacity; (**c**) dependence of adsorption capacity on density; (**d**) recyclability and stability of adsorption capacity of the new PU/CNT/NiFe_2_O_4_/PDMS sponge for various organic solvents and oil.

The separation efficiency of the prepared PU/CNT/NiFe_2_O_4_/PDMS sponge was studied by selectively separating chloroform/water, olive oil/water, acetone/water, hexane/water, toluene/water, gasoline/water, and ethanol/water mixtures. For this purpose, the PU/CNT/NiFe_2_O_4_/PDMS sponge was immersed in a mixture of oil, organic solvent, and water with a known composition until the organic liquids were completely absorbed. Then, the sponge was taken out of the water to measure the amount of oil or organic liquids remaining in the water. The separation efficiency of the prepared superhydrophobic sponge was calculated by measuring the amount of oil and organic liquids remaining in the water before and after absorption using Equation (1). Video S4 in the Supplementary Materials shows that after separation of the crude oil/water mixture, very little oil remained in the water, indicating high separation efficiency. In addition, the magnetic property makes it easy to control the PU/CNT/NiFe_2_O_4_/PDMS sponge in polluted waters. The results of the calculations of the separation efficiency of the PU/CNT/NiFe_2_O_4_/PDMS sponge, obtained for various mixtures, are presented in Figure 9a. The separation efficiencies of the newly prepared PU/CNT/NiFe_2_O_4_/PDMS sponge were 99.81, 99.27, 99.72, 99.59, 99.68, 99.59, and 99.74% for the chloroform/water, olive oil/water, acetone/water, hexane/water, toluene/water, gasoline/water, and ethanol/water mixtures, respectively. The effective separation properties of the obtained sponges can be explained by their high hydrophobicity and porosity, since small pore sizes cause strong capillary effects. These excellent properties of the PU/CNT/NiFe_2_O_4_/PDMS sponge make it a promising candidate for water purification from oil and organic compounds.

Secondly, we tested the absorption capacity of the newly prepared PU/CNT/NiFe_2_O_4_/PDMS sponge on oils and organic solvents. The experiment tested the sorption capacity of olive oil (density 0.918 g/mL) and six organic solvents, including chloroform, toluene, acetone, ethanol, gasoline, and hexane, with different densities of 1.49 g/mL, 0.867 g/mL, 0.786 g/mL, 0.789 g/mL, 0.710 g/mL, and 0.655 g/mL, respectively. Studies to determine the sorption capacity of the sponge were carried out by immersing a pre-weighed sponge in oil or organic solvents for 10 s. Oil or organic solvents impregnated in the PU/CNT/NiFe_2_O_4_/PDMS sponge were extracted by simple mechanical squeezing. To test for recyclability, the sponge was thoroughly dried in an oven, then dipped again in oil or organic solvents, and the absorbency was measured again. The absorption capacity (Q, g/g) of the new PU/CNT/NiFe_2_O_4_/PDMS sponge was calculated based on the difference between its masses before and after absorption using Equation (2). The initial mass of PU/CNT/NiFe_2_O_4_/PDMS sponge (m_1_) was 1.532 g. In order to accurately measure the absorption mass and avoid loss, the PU/CNT/NiFe_2_O_4_/PDMS sponge soaked in oil or organic solvent was immediately placed on a balance and weighed, and the experiments were performed three times to determine the average weight. This process was repeated for 5 cycles. The weighing results and average mass values of PU/CNT/NiFe_2_O_4_/PDMS after oil absorption (m_2_) are given in Table S1 (see Supplementary Material). According to the data given in Table S1 and using Equation (2), the absorption capacity of the resulting sponge was calculated. The results of calculating the absorption capacity (Q, g/g) are given in Table S2 (see Supplementary Materials) and shown in Figure 9b. The absorption capacity (Q, g/g) of the new PU/CNT/NiFe_2_O_4_/PDMS sponge ranged from 21.38 to 44.83 g/g (Figure 9b), highlighting the high absorption capacity [47]. The results showed a higher absorption capacity of the sponge in relation to chloroform (44.83 g/g); this is largely due to the different density (Figure 9c) and, to some extent, the volatility of organic solvents. Therefore, in Figure 9c, a noticeable decrease in the absorption capacity of the sponge can be seen with decreasing density of the tested oil and organic solvents. It is reported [28] that oil and organic solvents with a high density strongly adhere to hydrophobic materials through intermolecular hydrophobic interaction and thus exhibit high absorption capacity. This assumption is supported by the noticeable decrease in the absorption capacity of the sponge, which can be seen with a decrease in the density of the tested oil and organic solvents. A linear correlation was also found between the absorption capacity of the sponge and the density of the tested oil and organic solvents (Figure 9c) [48]. The process of testing the recyclability of the sponge was repeated for five cycles (with each cycle performed three times). The results show that the new PU/CNT/NiFe_2_O_4_/PDMS sponge absorbs oil and organic solvents 15–30 times its weight in 10 s of immersion, thereby demonstrating excellent absorption capacity (Figure 9d). After five cycles of use, the sorption capacity of the sponge for oil and organic solvents remained virtually unchanged, which indicates the high recyclability and mechanical resistance of the PU/CNT/NiFe_2_O_4_/PDMS sponge (Figure 9d) [29].

These results show that the sponge exhibits excellent absorption capacity for organic solvents and oils, and excellent retention of mechanical properties ensures high recyclability, so it can be concluded that the PU/CNT/NiFe_2_O_4_/PDMS sponge could become a promising and inexpensive material for practical use in purifying water contaminated with oil and organic solvents.

After the absorption process, the organic solvents extracted from the impregnated sponge were characterized based on appearance (color, odor), density, and refractive index. The results show that these characteristics of the organic solvents before and after the absorption tests remained unchanged, and the sponge did not lose its magnetic and hydrophobic properties after 10 cycles of use, indicating that there was no leaching of CNT, NiFe_2_O_4_, or PDMS and indicating the physical and chemical stability of the prepared PU/CNT/NiFe_2_O_4_/PDMS sponge.

**Figure 10:**
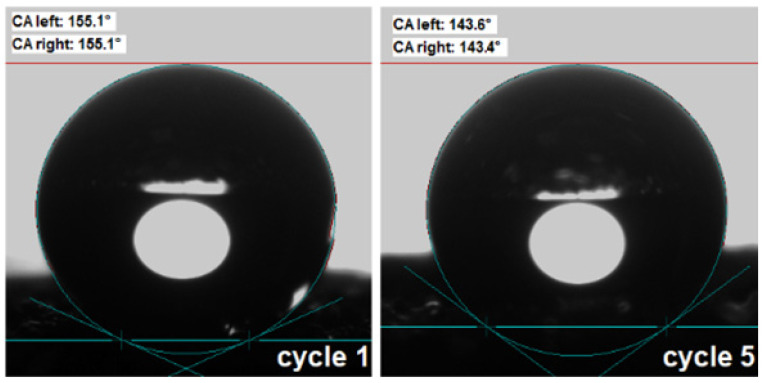
Water contact angle in 1st and 5th absorption cycles.

In addition, after five cycles of use, the PU/CNT/NiFe_2_O_4_/PDMS sponge retained its superhydrophobicity. Figure 10 shows the measurement of the water contact angle in one and five absorption cycles. The results show that the PU/CNT/NiFe_2_O_4_/PDMS sponge exhibited a superhydrophobic state after five cycles (after one cycle at 155.1° and five cycles at 143.6°).

The mechanical properties of the obtained PU/CNT/NiFe_2_O_4_/PDMS were determined by loading a 50 g weight for 10 min, the experiment was carried out in 10 repetitions, and each repetition was performed three times; the results obtained are presented in Figure S3. The structure and shape of the modified PU/CNT/NiFe_2_O_4_/PDMS sponge before and after a 50 g load remained completely unchanged. That is, after compression, the PU/CNT/NiFe_2_O_4_/PDMS sponge can easily return to its original shape several times, which proves the mechanical strength of the sponge wall. Also, during the sorption capacity test, the PU/CNT/NiFe_2_O_4_/PDMS sponge was repeatedly subjected to mechanical squeezing to separate the oil or organic solvents with which it was impregnated, which once again proves its mechanical stability.

**Figure 11:**
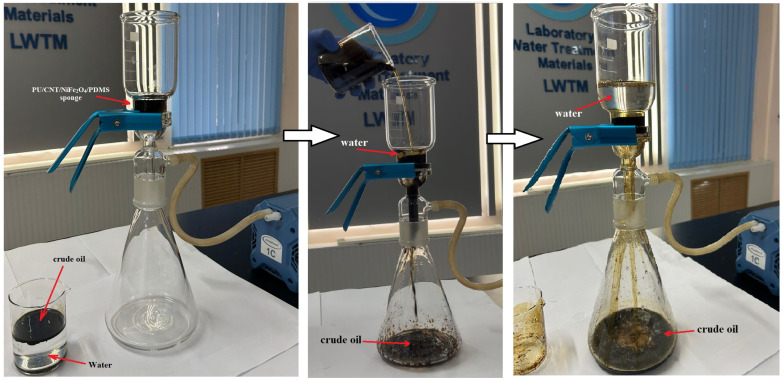
Continuous separation of oil and water from PU/CNT/NiFe_2_O_4_/PDMS sponge using glass vacuum filtration system.

The absorption and selective properties of the PU/CNT/NiFe_2_O_4_/PDMS sponge for the continuous separation efficiency of oil/water mixtures were also investigated. The study was conducted using a glass vacuum filtration system and the PU/CNT/NiFe_2_O_4_/PDMS sponge (Figure 11). The crude oil/water mixture with known mass composition was filtered through the glass vacuum system with the sponge installed therein. As shown in Figure 11, due to the continuous suction, the PU/CNT/NiFe_2_O_4_/PDMS sponge placed on the glass vacuum system quickly filtered the oil flowing through it, forming a crude oil stream gradually collected in the attached glass flask, and no water was detected in the collected crude oil [29,49]. In addition, there was almost no oil left on the surface of the water, and the separation efficiency of the crude oil/water mixture was approximately 99.5%. The percentage of separation efficiency was calculated based on the difference between the mass of the oil and water before and after separation (Figure 11). All experiments were performed in triplicate.

The obtained results show that the absorption capacity of the absorbents largely depends on the immersion time and the physical properties of the absorbed substance (density, surface tension). Also, the test results show that the PU/CNT/NiFe_2_O_4_/PDMS sponge exhibits high separation efficiency of oil and organic solvent/water mixtures (up to 99.81%) and fast (immersion time 10 s) and excellent absorption capacity (from 21.38 to 44.83 g/g) for oil and organic solvents, as well as superhydrophobicity (water absorption of about 0.4%) [49]. A comparison of the absorption capacity of PU/CNT/NiFe_2_O_4_/PDMS with many other superhydrophobic sorbent composites, including sorbents displaying magnetic properties, described in the literature (Table 1) indicates the high potential of the PU/CNT/NiFe_2_O_4_/PDMS sponge for practical applications for cleaning up oil spills and removing organic pollutants from water surfaces and its competitiveness.

## 4. Conclusions

In this work, we reported the successful synthesis of CNTs through chemical vapor deposition (CVD) on alumina- supported nickel ferrite (NiFe_2_O_4_/Al_2_O_3_) catalyst, as demonstrated by TEM and XRD results. The synthesis of magnetic nickel ferrite (NiFe) nanosized particles was also reported using the sol–gel method. XRD allowed to identify the spinel structure and determine the crystal size (21–29 nm), indicating the formation of the desired nanostructure. Magnetic characterization showed that NiFe particle is highly magnetic (43 Am^2^kg^−1^), with a low coercivity (93 Oe) and remanence. This magnetic characteristic of the NiFe nanoparticle (and also the CNTs) is a critical characteristic to improve the practical application of the sponges designed in this work.

This paper describes the facile fabrication of a new superhydrophobic/oleophilic magnetic PU/CNT/NiFe_2_O_4_/PDMS sponge based on CNTs, NiFe_2_O_4_ magnetic nanoparticles, and PDMS with the immersion coating method used for the PU sponge. The PU/CNT/NiFe_2_O_4_/PDMS sponge was characterized using SEM, water contact measurements (>150°), and videos of water and oil droplets on the sponge surface. Also, the efficiency of selective separation, the sorption capacity, and the possibility of recycling in the purification of wastewater from oil and organic solvents were studied. These studies showed that the PU/CNT/NiFe_2_O_4_/PDMS sponge exhibited great superhydrophobic properties, mechanical strength, and stability, high separation efficiency, and excellent absorption capacity (21–45 g/g). The PU/CNT/NiFe_2_O_4_/PDMS sponge has the ability to rapidly (in approximately 10 s) and selectively absorb different types of oils and organic liquids. Thus, it can be concluded that the superhydrophobic and superoleophilic PU/CNT/NiFe_2_O_4_/PDMS sponge will find practical applications in the future, such as for removing oil spills using actual oily wastewater (including that containing salts and emulsifiers) and lipophilic organic pollutants from water surfaces. In addition, the results reported here open up the possibility of fabricating PU sponges coated with CNTs from actual plastic waste, which should further contribute to avoiding pollution in water surfaces.

## Figures and Tables

**Table 1:** A comparison of the absorption capacities of the different sorption materials described in the literature.

Absorbents	Organics	Q, g/g	References
PU/CNT/NiFe_2_O_4_/PDMS	Crude oil, olive oil, chloroform, toluene, acetone, ethanol, gasoline, hexane	21.38–44.83	In this work
CNT/PDMS-coated PU sponge	Soybean oil, used motor oil, diesel oil, n-hexadecane, gasoline, n-hexane	15–25	[50]
Fe_3_O_4_-PDMS/MWNTs sponge	Dichloromethane, petroleum ether, hexane, chloroform, tetrahydrofuran, toluene, gasoline	8.5–20	[51]
PDMS@Fe_3_O_4_/MS sponge	kerosene, diesel, corn germ oil	14.68–17.99	[52]
Fe_3_O_4_@carbon aerogel	Engine oil, chloroethane, corn oil	10–11	[53]
PU/MgFe_2_O_4_/RGO/SO sponge	Crude oil, olive oil, chloroform, toluene, ethanol, acetone, hexane	16.61–44.86	[28]
PU/MgFe_2_O_4_/SO sponge	Olive oil, chloroform, toluene, ethanol, acetone, hexane	3.5–19	[28]
ZIF-8/rGO/PU foam	Chloroform, hexane, acetonitrile, toluene, acetone, methanol, ethanol, isopropyl alcohol, butanol, octanol, ethylene glycol	15–35	[54]

## Data Availability

Data is contained within the article or Supplementary Materials.

## Supplementary Materials

The following supporting information can be downloaded at: https://www.mdpi.com/article/10.3390/nano14231942/s1, Figure S1: CVD reactor used to synthesize carbon nanotubes; Figure S2: Linear regression to obtain crystallite size using (a) Halder–Wagner and (b) size–strain plot mathematical models; Figure S3: Photos of testing the mechanical properties of the PU/CNT/NiFe2O4/PDMS sponge; Table S1: The mass of the PU/CNT/NiFe2O4/PDMS sponge after 10 s of immersion in Olive oil and various organic solvents; Table S2: Absorption capacity of PU/CNT/NiFe2O4/PDMS sponge for olive oil and various organic solvents; Text S1: Determination of crystallite size of NiFe2O4 from XRD data. Video S1: Magnetic properties of sponge; Video S2: Hydrophobic properties; Video S3: Absorbtion of petrolium; Video S4: Wateroil separation.
