# Supplementary material for: Carbon-Nanotube-Based Superhydrophobic Magnetic Nanomaterial as Absorbent for Rapid and Efficient Oil/Water Separation
Source: Nanomaterials (Basel). 2024 Dec 3;14(23):1942. doi: 10.3390/nano14231942 (PMC11643525; doi:10.3390/nano14231942)
Supplement: Supplementary file 1 [file nanomaterials-14-01942-s001.zip › nanomaterials-3331236-supplementary.pdf]

Supplementary file

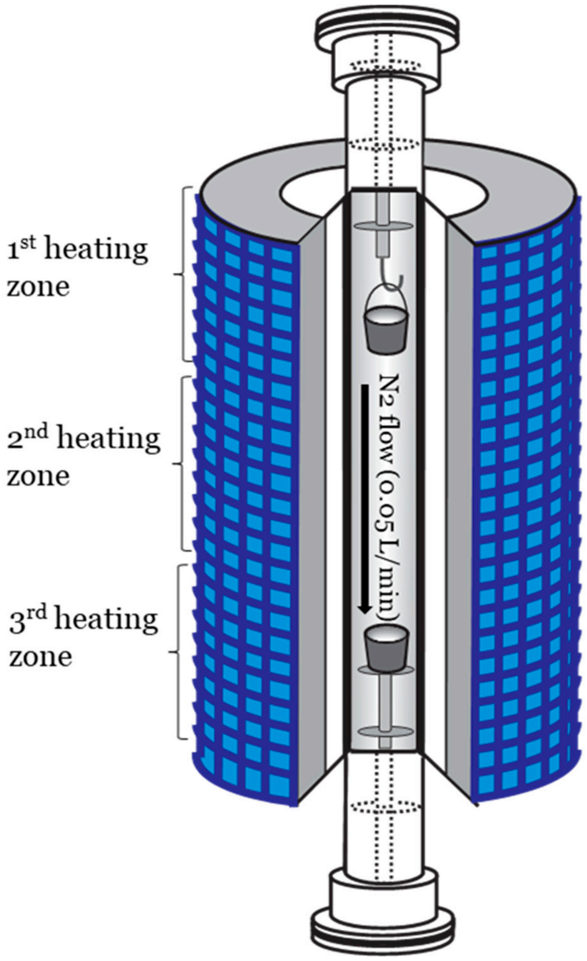

Figure S1. CVD reactor used to synthesize carbon nanotubes.

Text S1. Determination of crystallite size of $NiFe_2O_4$ from XRD data

The XRD data that correspond to the nanoparticle $NiFe_2O_4$ were used to estimate the crystallite size using two mathematical approaches, the Halder–Wagner method and a size–strain plot, following the approach described by Nath et al. [37].

The Halder–Wagner approach is represented by Equation (1):

$$\left(\frac{\beta_{hkl}^{*}}{d_{hkl}^{*}}\right)^{2} = \frac{1}{D}\cdot\frac{\beta_{hkl}^{*}}{{d_{hkl}^{*}}^{2}} + \left(\frac{\epsilon}{2}\right)^{2} \qquad (1)$$

where $\beta_{hkl}^{*} = \beta_{hkl} \cdot \cos(\theta)$ and $d_{hkl}^{*} = 2 \cdot d_{hkl} \cdot \sin(\theta)$.

In the above equation, β_{hkl} is the full width at half of the maximum intensity, D is the average crystallite size, ε is the strain induced in powders, and d^{*}_{hkl} is the lattice distance between the (hkl) planes and for the cubic crystal. By plotting $\frac{\beta_{hkl}^{*}}{{d_{hkl}^{*}}^{2}}$ on the X-axis versus $\left(\frac{\beta_{hkl}^{*}}{d_{hkl}^{*}}\right)^{2}$ on the Y-axis, it is possible to determine the average crystallite size based on the slope of the curve generated.

The size–strain approach is represented by Equations (2) and (3):

$$(d_{hkl} \cdot \beta_{hkl} \cdot \cos\theta)^{2} = \frac{k\lambda}{D} \cdot (d_{hkl}^{2} \cdot \beta_{hkl} \cdot cos\theta) + \frac{\epsilon^{2}}{4} \qquad (2)$$

$$d_{hkl}^{2} = \frac{a^{2}}{h^{2} + l^{2} + k^{2}} \qquad (3)$$

In which d_{hkl} is the lattice distance between (hkl) planes for the cubic crystal, β_{hkl} is the full width at half of the maximum intensity, θ is the refraction angle, k is the shape factor, D is the average crystallite size, and ε is the strain induced in the powders. The average crystallite size is obtained based on the slope of the curve plotted by $(d_{hkl}^{2} \cdot \beta_{hkl} \cdot cos\theta)$ on the X-axis

and $(d_{hkl} \,.\, \beta_{hkl} . \cos\theta)^2$ on the Y-axis. The linear regression obtained for calculating the crystallite size for $NiFe_2O_4$ for each methodology is shown in Figure S2.

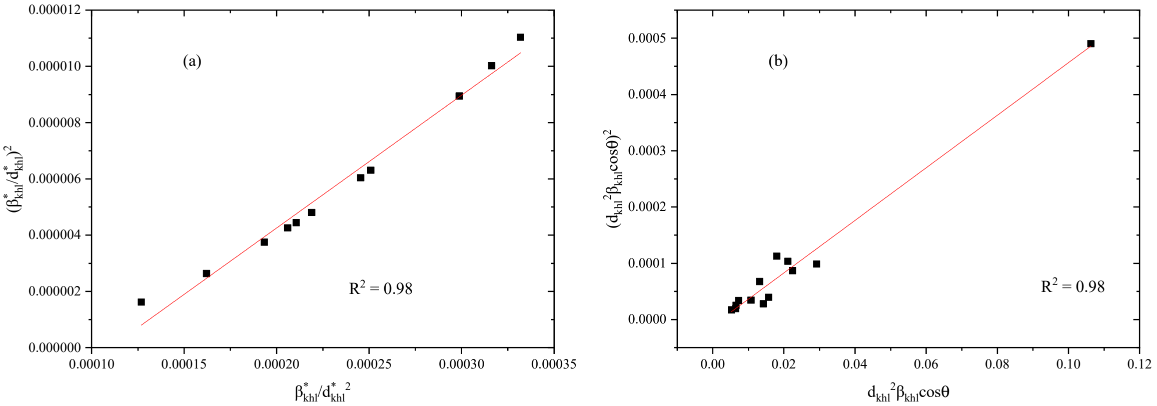

Figure S2. Linear regression to obtain crystallite size using (a) Halder–Wagner and (b) size–strain plot mathematical models.

Table S1. The mass of the PU/CNT/$NiFe_2O_4$/PDMS sponge after 10 seconds of immersion in Olive oil and various organic solvents.

	Absorption capacity (Q, g)									
**Cycle**	**1**		**2**		**3**		**4**		**5**	
Chloroform	70.10		71.97		68.66		70.84		68.97	
(1.49 g/cm^3)	71.35	**70.75**	71.05	**71.16**	70.36	**68.67**	70.33	**70.84**	70.84	**69.65**
	70.79		70.46		66.98		71.35		69.14	
Olive oil	49.28		49.61		50.51		48.44		51.77	
(0.918 g/cm^3)	49.54	**49.06**	47.71	**48.39**	48.84	**49.91**	49.12	**48.59**	50.39	**50.24**
	48.35		47.84		50.39		48.21		48.58	
Toluene	46.99		48.00		47.05		46.53		50.26	
(0.867 g/cm^3)	44.46	**46.00**	46.11	**47.12**	47.74	**46.83**	44.47	**46.18**	48.69	**48.65**
	46.54		47.25		45.70		47.55		46.99	
Ethanol	45.38		45.94		45.39		45.22		48.76	
(0.789 g/cm^3)	45.90	**45.99**	45.06	**45.39**	45.68	**45.23**	47.97	**46.19**	45.91	**46.74**
	46.70		45.18		44.63		45.39		45.53	
Acetone	43.94		38.82		44.40		43.72		44.15	
(0.786 g/cm^3)	44.69	**44.11**	43.49	**41.31**	43.68	**43.83**	38.73	**41.91**	43.85	**43.59**
	43.69		41.62		43.40		43.28		42.76	
Gasoline	40.32		42.19		38.58		41.93		42.05	
(0.710 g/cm^3)	38.55	**40.19**	39.79	**41.31**	41.82	**40.11**	38.59	**40.77**	42.82	**42.28**
	41.72		41.96		39.94		41.79		41.96	
Hexane	34.04		33.49		32.46		33.35		39.27	
(0.655 g/cm^3)	33.14	**33.57**	35.19	**33.84**	30.85	**32.38**	38.27	**35.02**	36.94	**36.66**
	33.52		32.83		33.81		33.43		33.77	

Table S2. Absorption capacity of PU/CNT/$NiFe_2O_4$/PDMS sponge for olive oil and various organic solvents.

Absorption capacity (Q. g/g)										
**Cycle**		**1**		**2**		**3**		**4**		**5**
Chloroform (1.49 g/cm^3)	44.76	**45.18**	45.98	**45.45**	43.82	**43.82**	45.24	**45.24**	44.02	**44.46**
	45.57		45.38		44.93		44.91		45.24	
	45.21		44.99		42.72		45.57		44.13	
Olive oil (0.918 g/cm^3)	31.17	**31.02**	31.38	**30.58**	31.97	**31.58**	30.62	**30.72**	32.79	**31.80**
	31.34		30.14		30.88		31.06		31.89	
	30.56		30.23		31.89		30.47		30.71	
Toluene (0.867 g/cm^3)	29.67	**29.02**	30.33	**29.76**	29.71	**29.57**	29.37	**29.15**	31.81	**30.75**
	28.02		29.10		30.16		28.03		30.78	
	29.38		29.84		28.83		30.04		29.67	
Ethanol (0.789 g/cm^3)	28.62	**29.02**	28.99	**28.63**	28.63	**28.53**	28.52	**29.15**	30.83	**29.51**
	28.96		28.41		28.82		30.31		28.97	
	29.48		28.49		28.13		28.63		28.72	
Acetone (0.786 g/cm^3)	27.68	**27.79**	24.34	**25.97**	27.98	**27.61**	27.54	**26.36**	27.82	**27.45**
	28.17		27.39		27.51		24.28		27.62	
	27.52		26.17		27.33		27.25		26.91	
Gasoline (0.710 g/cm^3)	25.32	**25.24**	26.54	**25.97**	24.18	**25.18**	26.37	**25.61**	26.45	**26.60**
	24.16		24.97		26.30		24.19		26.95	
	26.23		26.39		25.07		26.28		26.39	
Hexane (0.655 g/cm^3)	21.22	**20.91**	20.86	**21.09**	20.19	**20.13**	20.77	**21.86**	24.63	**22.93**
	20.63		21.97		19.14		23.98		23.11	
	20.88		20.43		21.07		20.82		21.04	

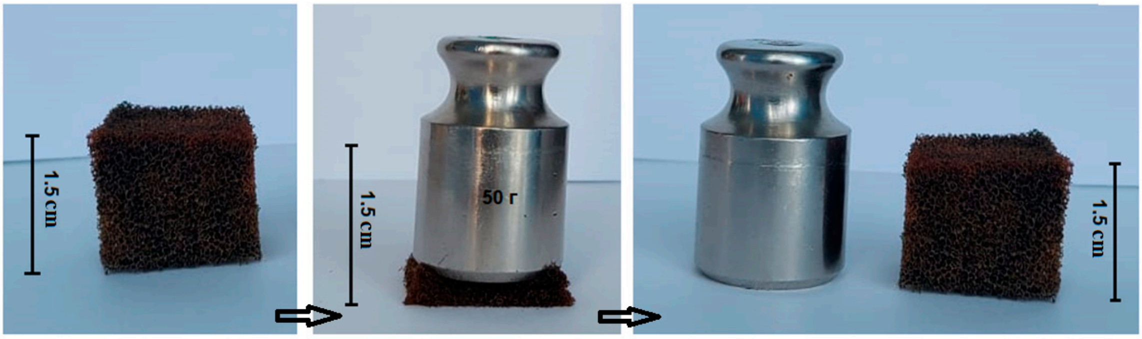

Figure S3. Photos of testing the mechanical properties of the PU/CNT/$NiFe_2O_4$/PDMS sponge.
